# Cellular Responses and Tissue Depots for Nanoformulated Antiretroviral Therapy

**DOI:** 10.1371/journal.pone.0145966

**Published:** 2015-12-30

**Authors:** Andrea L. Martinez-Skinner, Mariluz A. Araínga, Pavan Puligujja, Diana L. Palandri, Hannah M. Baldridge, Benson J. Edagwa, JoEllyn M. McMillan, R. Lee Mosley, Howard E. Gendelman

**Affiliations:** 1 Department of Pharmacology and Experimental Neuroscience, University of Nebraska Medical Center, Omaha, NE, 68198–5880, United States of America; 2 Department of Internal Medicine, University of Nebraska Medical Center, Omaha, NE, 68198–5880, United States of America; Commissariat a l'Energie Atomique(cea), FRANCE

## Abstract

Long-acting nanoformulated antiretroviral therapy (nanoART) induces a range of innate immune migratory, phagocytic and secretory cell functions that perpetuate drug depots. While recycling endosomes serve as the macrophage subcellular depots, little is known of the dynamics of nanoART-cell interactions. To this end, we assessed temporal leukocyte responses, drug uptake and distribution following both intraperitoneal and intramuscular injection of nanoformulated atazanavir (nanoATV). Local inflammatory responses heralded drug distribution to peritoneal cell populations, regional lymph nodes, spleen and liver. This proceeded for three days in male Balb/c mice. NanoATV-induced changes in myeloid populations were assessed by fluorescence-activated cell sorting (FACS) with CD45, CD3, CD11b, F4/80, and GR-1 antibodies. The localization of nanoATV within leukocyte cell subsets was determined by confocal microscopy. Combined FACS and ultra-performance liquid chromatography tandem mass-spectrometry assays determined nanoATV carriages by cell-based vehicles. A robust granulocyte, but not peritoneal macrophage nanoATV response paralleled zymosan A treatment. ATV levels were highest at sites of injection in peritoneal or muscle macrophages, dependent on the injection site. The spleen and liver served as nanoATV tissue depots while drug levels in lymph nodes were higher than those recorded in plasma. Dual polymer and cell labeling demonstrated a nearly exclusive drug reservoir in macrophages within the liver and spleen. Overall, nanoART induces innate immune responses coincident with rapid tissue macrophage distribution. Taken together, these works provide avenues for therapeutic development designed towards chemical eradication of human immunodeficiency viral infection.

## Introduction

Human immunodeficiency virus (HIV) therapeutics have consistently evolved over the past three decades as newer antiretroviral (ARV) medicines have come on-line and have demonstrated improved bioavailability, antiviral responses, ease of administration, and reduced toxicities [1–6]. An unmet need for HIV/AIDS patient care rests in the development of long acting ARVs towards improving drug adherence and in the ability to better target viral cell and tissue reservoirs of infection [7–9]. This includes specific viral growth sites in lymph nodes, gut and brain with coincident extensions of drug half-life [8, 10, 11]. Improved targeting of sites of viral infection was shown by establishing drug depots in mononuclear phagocytes (MP; monocytes and macrophages) made possible by targeted nanoparticle cell delivery and consequent slow release of the ARV at disease sites [12–19]. Notably, facilitating such depots of ARV can speed reduction of residual virus and lower viral transmission, dissemination, resistance and end organ disease [20–23]. The ultimate elimination of infection by “chemical cure” is possible with long-acting ARV that effectively prolongs the interval for ARV administration. Our laboratory has embraced such challenges through the development of injectable MP-targeted nanoformulated antiretroviral therapies (nanoART) [14, 17, 18, 20, 23–26]. The nanoformulation of crystalline drugs with poor solubility has enabled extended ARV release and provided attractive alternatives to oral drug administration [17–19, 23].

Such prior works, however, have centered on MP cell culture assays for drug uptake, release and retention, or alternatively on pharmacokinetic (PK) analyses [12, 13, 17, 20, 24, 25]. The engagement of nanoART with the innate immune system and its subsequent effect on drug biodistribution has not yet been elucidated. To such ends, we administered nanoformulated atazanavir (nanoATV), a long-acting ARV, to Balb/c mice. At various times after injection, cellular immune profiles, carrying capacities, and drug biodistribution were determined. Tissue drug depots and identification of cellular depots were examined. The results demonstrated that macrophages are the major cells that take up nanoART following intraperitoneal (IP) administration. Furthermore, tissue macrophages were the principal, if not sole reservoir for the nanoparticles with rapid and sustained ARV lymphatic targeting. These data, taken together, support a central role for the macrophage as a carrier of nanoART to sites of viral infection.

## Materials and Methods

### NanoATV Preparation

ATV-sulfate was purchased from Gyma Laboratories of America, Inc. (Westbury, NY) and the free base form was made using 1N NaOH. The surfactant used for the formulation generation was poloxamer-188 (P188; Sigma-Aldrich, St. Louis, MO) or CF633-labeled P188. CF633-labeled P188 was synthesized by conjugating CF633 (Biotium, Hayward, CA) to the P188 polymer as described previously [13]. For nanoformulation preparation free-base drug (1.0% by weight) and polymer (0.5% by weight) were mixed in 10 mM HEPES buffer (Sigma-Aldrich, St. Louis, MO), pH 7.8, in a volume of 15 ml. Homogeneous suspensions were prepared at room temperature using an Avestin C3 high-pressure homogenizer (Avestin Inc, Ottawa, ON) by passaging the suspension at 20,000 psi until the desired particle size (300–400 nm) was attained, as described [25]. Particle size, polydispersity (PDI) and surface charge (zeta potential) of the nanosuspension were determined by dynamic light scattering using a Malvern Zetasizer Nano Series Nano-ZS (Malvern Instruments Inc, Westborough, MA). Once a size of 200–300 nm, charge of ^-^20 to ^-^30 mV, and a PDI of 0.2 were achieved, the nanosuspension was centrifuged at 10,000 x g for 30 min at 4°C; the resulting pellet was resuspended in surfactant solution (0.5% P188) containing 9.25% sucrose to adjust tonicity and lyophilized. Drug content of the CF633-labeled P188 nanosuspension and lyophilized P188-ATV nanoformulation (0.32 mg ATV/mg lyophilized nanoformulation) were determined by reversed-phase high performance liquid chromatography [26]. Lyophilized nanoformulation was resuspended in phosphate buffered saline (PBS) for treatment.

### Animals and ARV Treatment

Male Balb/c mice (8–10 wk old) were purchased from Jackson Laboratories (Bar Harbor, ME). Mice were maintained on standard rodent chow and water, ad libitum, in animal facilities illuminated on a 12/12 h light/dark cycle. All animal work was carried out in accordance with the Guide for the Care and Use of Laboratory Animals as adopted by the National Institutes of Health under a protocol approved by the University of Nebraska Medical Center Institutional Animal Care and Use Committee (IACUC). Mice were injected IP or intramuscularly (IM) with 100 mg/kg nanoATV, 0.5% P188, or 0.1 mg of zymosan A in 100 μl PBS for IP or 50 μl PBS for IM administration. For tissue collection, mice were anesthetized by isoflurane inhalation and blood and tissues removed according to the approved IACUC protocol.

### Collection of Peritoneal Cells and Fluids

Peritoneal lavages were collected 1, 4, 12, 24, 48, and 72 h after treatment. The mice were anesthetized using isoflurane and cells were collected by peritoneal lavage with 3 mL cold PBS containing 3% bovine serum albumin (BSA) (i.e., fluorescence-activated cell sorting (FACS) buffer) followed by gentle massage of the abdomen and aspiration with a syringe. The peritoneal fluid was then centrifuged at 860 x g for 8 min at 4°C. Supernatants were stored at -20°C until further analysis. Cell pellets were washed once in 5 mL of FACS buffer and re-suspended in 1 mL of buffer for further FACS analysis [27, 28].

### FACS Analyses

Resuspended cell pellets were incubated in a solution of immunoglobulin from murine serum at a 1:500 concentration for 15 min. Cells were centrifuged at 860 x g for 8 min at 4°C and resuspended in 1 mL of FACS buffer. Cells were then incubated with 1.0 μg/mL of various monoclonal antibodies for 30 min at room temperature. Fluorescently labeled antibodies were used for the detection of cell surface markers. The antibodies included PECy7-CD11b (BD Pharmingen, San Jose, CA), PE-F4/80 (Life Technologies-Invitrogen, Grand Island, NY), and eFluor660-Ly6C/Gr-1 (eBioscience, San Diego, CA) or PECy7-CD45 (Biolegend, San Diego, CA), FITC-CD3 (BD Biosciences, San Jose, CA), PE-CD11b (eBioscience, San Diego, CA) and BV421-F4/80 (BD Biosciences, San Jose, CA) for testing samples after IP or IM injections, respectively. For FACS analyses, a portion of cells were isolated and used as unstained controls. In addition, compensation beads and unstained cells were incubated individually with each antibody for 30 min (assay controls). After such incubations, cells were washed with FACS buffer, then centrifuged at 860 x g for 8 min at 4°C and re-suspended in buffer. Isolated and stained peritoneal cells were sorted by FACS on a BD FACSAria I cell sorter using the FACS DIVA version 6 software (BD Immunocytometry Systems, San Jose, CA). Debris and lymphocytes were excluded from the analysis using forward and side scatter gating. Cells were then gated based on CD11b and Ly6C/GR-1. The CD11b negative and Ly6C/GR-1 negative cell populations were sorted as the unstained cell population. CD11b^mid^ Ly6C/Gr-1 negative cells were sorted as the differentiating cell population. CD11b^high^ cells were then gated for F4/80. F4/80 positive cells were sorted as macrophages and F4/80 negative cells were sorted as granulocytes. Back gating was used to ensure the GR-1 high status of F4/80 negative cells. Tissue samples after IM injection were also collected and analyzed by flow cytometry for the identification of CD45+CD3+ (lymphocytes), CD45+CD3-CD11b+F4/80+ (macrophages) and CD45+CD3-CD11b+F4/80- (granulocytes). Flow cytometric analysis was performed using using FlowJo® software.

### ATV Quantitation

For nanoATV treated mice, peritoneal cells isolated by FACS were centrifuged at 20,800 x g for 10 min. Supernatants were removed and cell pellets stored at -80°C until further processing. Cell pellets were re-suspended in 50 μL of PBS and vortexed for 3 min. Cell lysates were then processed for drug quantitation by ultra-performance liquid chromatography tandem mass-spectrometry (UPLC-MS/MS) [29]. Plasma, liver, spleen, and lymph nodes were collected from nanoATV treated mice and stored at -80°C until analysis of drug content.

### Confocal Microscopy

Tissues collected from mice treated with CF633-labeled nanoATV (liver, spleen, mesenteric lymph nodes or muscle) were flash-frozen in Tissue-Tek O.C.T. compound (Thermo Fisher Scientific, Waltham, MA) using dry ice. Following cryogenic sectioning, slides were fixed in 3.7% formaldehyde for 15–20 min followed by a PBS wash. Slides were then incubated in 0.5% Triton X-100 for 5–7 min followed by a PBS wash. To control for non-specific binding, slides were blocked for 30 min in 5% BSA followed by a PBS wash. Slides were incubated with rat anti-F4/80 (Abcam, Cambridge, MA) or rabbit anti-CD68 (Abcam, Cambridge, MA) at a 1:100 dilution in 2.5% BSA for 1 h and washed 3 times with PBS. Slides were then incubated with Alexa Fluor 488 goat anti-rat (Life Technologies-Molecular Probes, Grand Island, NY) or Alexa Fluor 488 chicken anti-rabbit (Life Technologies-Molecular Probes, Grand Island, NY) at a 1:100 dilution in 2.5% BSA for 1 h followed by three PBS washes. ProLong Gold anti-fade reagent with DAPI (Thermo Fisher Scientific, Waltham, MA) was used to adhere a coverslip. Slides were evaluated with a Zeiss LSM710 confocal microscope using Zen 2011 software (Carl Zeiss Microimaging Inc., Thornwood, NY). Additional spleen and lymph node sections were stained with hematoxylin and eosin (H&E) and images obtained with a Nuance EX camera fixed to a Nikon Eclipse E800 using Nuance software (Cambridge Research & Instrumentation, Woburn, MA).

### Statistical Analyses

One-way analysis of variance (ANOVA) was used to assess significant differences amongst cell populations, drug-particle distribution, and plasma/tissue drug distribution and variation unless otherwise indicated. Extreme outliers beyond the 99% confidence interval of the mean and 3-fold greater than the SEM were excluded. Student’s t-test was used for determining the differences between macrophage, lymphocyte and granulocyte cell populations in spleen following IM injection. Factorial ANOVA was used to determine differences in ATV concentration per 10^6^ cells for each cell type isolated from peritoneal lavage over time after drug administration. Pair-wise comparisons of the means from 4–8 mice per group were evaluated by Fisher's least significant difference post-hoc tests.

## Results

### NanoATV Immune Responses

P188-ATV and CF633-P188-ATV were prepared as described in our previous publications [13, 18–20, 30]. Lyophilized formulations were resuspended in PBS with vortexing to achieve an injection suspension of 25 or 50 mg ATV/ml for IP or IM, respectively. The particle size of either formulation upon resuspension was 300–400 nm with a PDI of 0.25–0.3 and zeta potential of -25 to -30 mV.

**Fig 1:**
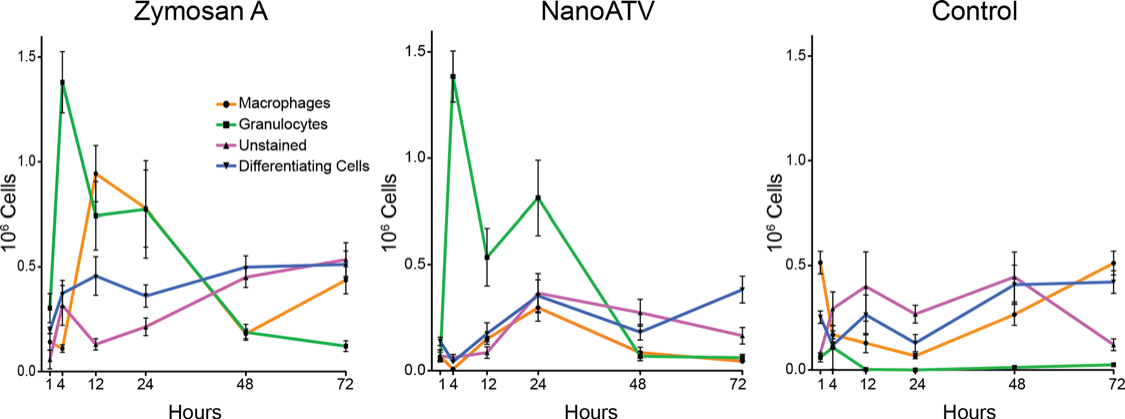
Differential cell counts in peritoneal lavage. Following intraperitoneal injection of zymosan A, nanoATV, or P188 (control), cells were collected by peritoneal lavage at 1, 4, 12, 24, 48, and 72 h. Collected cells were stained with antibodies directed against GR-1, CD11b, and F4/80 and sorted by FACS. Mean total cell numbers per mouse ± SEM were calculated from 4–8 mice/treatment group/time point.

We posited that nanoART elicits a similar activation state of macrophages *in vivo* as observed in cultures of human monocyte-derived macrophages without substantial inflammatory responses [30]. To test this hypothesis, we adapted a peritonitis model to quantitatively assess the biological and cellular responses *in vivo* [31, 32]. To assess the temporal and differential cellular responses following treatment, we examined the cellular environment of the peritoneum using FACS for granulocytes (CD11b^high^, GR-1^high^), mature macrophages (CD11b^high^, F4/80 positive cells), immature macrophages (differentiating cells) (CD11b^med^, GR-1 negative), and an unstained cell population (CD11b negative, GR-1 negative) [28, 31–35]. We examined cellular responses over a 72 h period after IP injection with P188 (the polymer used in nanoATV preparation) or zymosan A (a known inflammatory reagent) and compared these findings to responses elicited by 100 mg/kg of nanoATV (Fig 1).

Similar to a traditional inflammatory response, nanoATV recruited granulocytes into the peritoneum following injection. Fig 1 shows the granulocyte population reached a maximum 4 h after nanoATV administration and then returned to normal by 48 h. This was similar to the response following zymosan A treatment; however, granulocyte infiltration following zymosan A treatment occurred earlier (1 h) and return to control level was not achieved until 72 h. The polymer control did not induce a granulocyte response suggesting that the vehicle alone did not induce an inflammatory response.

NanoATV did not increase macrophage numbers following IP administration. Significantly fewer macrophages were found within the peritoneum at nearly all time points compared to zymosan A treatment. The macrophage cell population decreased within 1 h following nanoATV treatment reaching a minimum at 4 h before modest transient increase at 24 h. Macrophage numbers had not returned to control levels by 72 h. Parallel to nanoATV, zymosan A reduced the macrophage cell population within 1 h. However, by 4 h macrophage numbers rebounded to levels higher than observed for P188 or nanoATV treatment, reaching a maximum at 12 h, after which numbers again declined before returning to normal levels at 72 h. P188 treatment reduced macrophage numbers after 1 h reaching a nadir by 24 h followed by a rebound of macrophage numbers by 72 h.

The differentiating cell population within the peritoneum varied between 100,000 to 500,000 cells at all times following nanoATV, zymosan A and P188 treatment. All treatments resulted in similar numbers of differentiating cells by 72 h. The unstained cell population also varied minimally with time in all treatment groups. This cell population peaked 24 h after nanoATV treatment with gradual reductions in numbers thereafter.

### Peritoneal Cell Drug Depots

**Fig 2:**
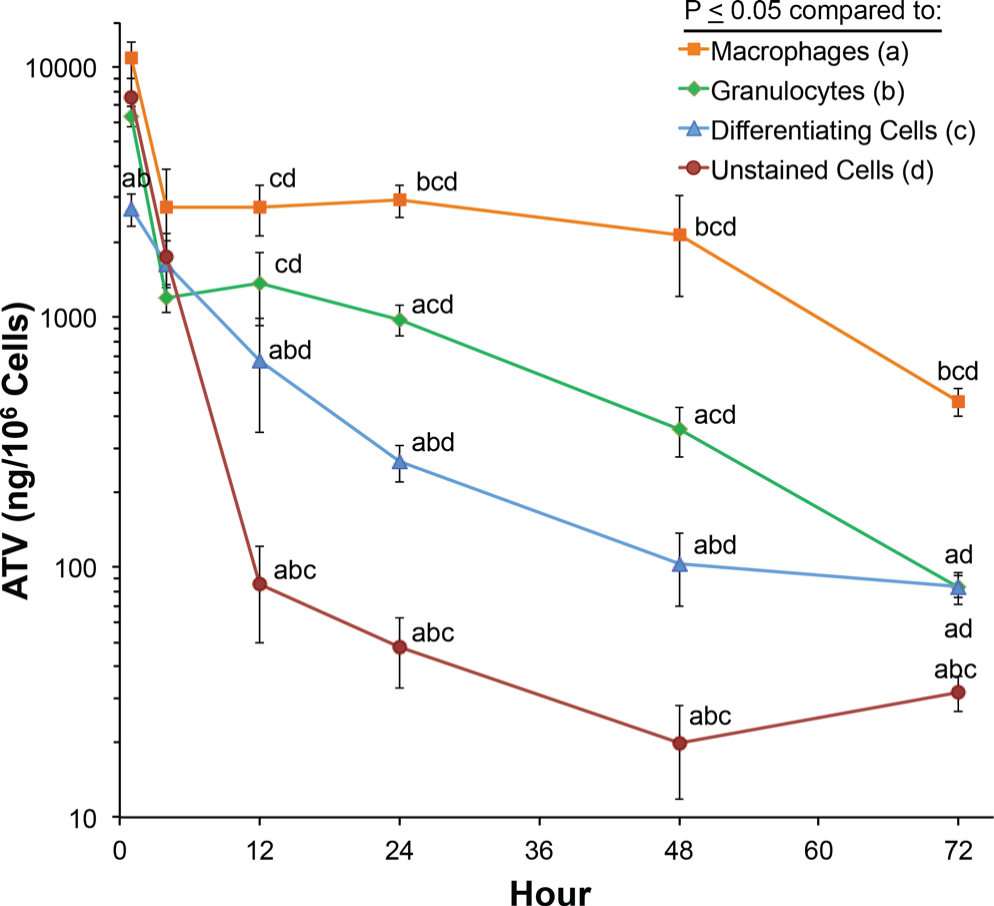
Cellular ATV concentrations. Following peritoneal injection of nanoATV, cellular exudates were collected via peritoneal lavage 1, 4, 12, 24, 48 and 72 h post injection. Cells were then probed with GR-1, CD11b, and F4/80 antibodies and sorted via FACS. NanoATV concentrations were determined in cellular fractions by UPLC-MS/MS. Cell fractions consisted of granulocytes (CD11b^high^, GR-1 positive cells), mature macrophages (CD11b^high^, F4/80 positive cells), immature macrophages/differentiating cells (CD11b^med^), and an unstained cell population. Mean cell drug levels ± SEM are reported for 4–8 mice/group/time point.

We posited that macrophages would be the major *in vivo* cellular nanoART depot. To test this hypothesis, we assessed drug concentrations within individual FACS-sorted peritoneal cell populations over the course of 72 h following nanoATV administration. Within 1 h following nanoATV administration, drug was found within differentiating cells, granulocytes, unstained cells, and macrophages (Fig 2). Drug concentrations reached 3,000–11,000 ng ATV/10^6^ cells and the highest levels were within macrophages. Drug concentrations in differentiating cells gradually decreased over 48 h and then stabilized over the next 24 h. Drug concentration in unstained cells declined rapidly over the initial 12 h post nanoATV treatment, but remained well above the limit of quantitation thereafter. Drug concentrations within granulocytes and macrophages rapidly decreased over the initial 4 h, but declined more slowly beginning at 12 h with relative constant decline thereafter. By 72 h, drug levels in all cell groups except macrophages were significantly reduced to < 100 ng/10^6^ cells. Such reduction was likely due to cell turnover and regional tissue distribution. In contrast, macrophages retained nearly 500 ng ATV/10^6^ cells at 72 h.

### Tissue Drug Depots

**Fig 3:**
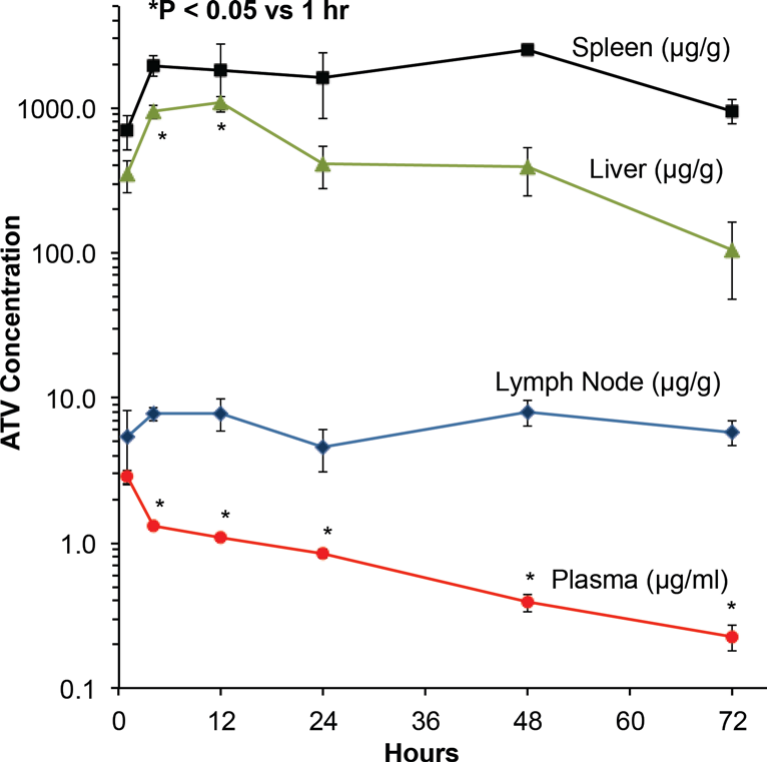
ATV concentrations in plasma, lymph nodes, liver and spleen. Following intraperitoneal injection of nanoATV, plasma, mesenteric lymph nodes, liver and spleen were collected 1, 4, 12, 24, 48, and 72 h post injection. ATV concentrations were determined by UPLC-MS/MS. Tissue drug levels are reported as mean ± SEM for 4–8 mice/time point.

We posited that tissues containing high numbers of macrophages would be major *in vivo* tissue nanoART depots. To test this hypothesis, we determined tissue ATV concentrations up to 72 h after nanoATV administration in mesenteric lymph nodes, liver, and spleen. Drug concentrations in plasma peaked 1 h post-injection, but declined thereafter (Fig 3). There was an initial rapid decline in plasma drug concentrations over 4 h presumably due to metabolism and/or deposition of drug within tissues and a more gradual decline thereafter. By 72 h after injection, plasma drug levels had declined to the limit of quantitation (0.2 ng/ml). ATV concentrations were seen in lymph nodes within one hour and levels were maintained over 72 h with an average overall drug concentration between 5 and 8 μg ATV/g tissue. Maximal drug concentration within the liver was achieved at 12 h post injection at 1200 μg ATV/g tissue. By 72 h, 100 μg ATV/g tissue remained within liver. Similar to liver, high levels of ATV were detected in spleen by 4 h post-treatment. However, in contrast to liver, drug concentrations in spleen were maintained over the 72 h observation period.

### Cellular Drug Depots in Tissues

**Fig 4:**
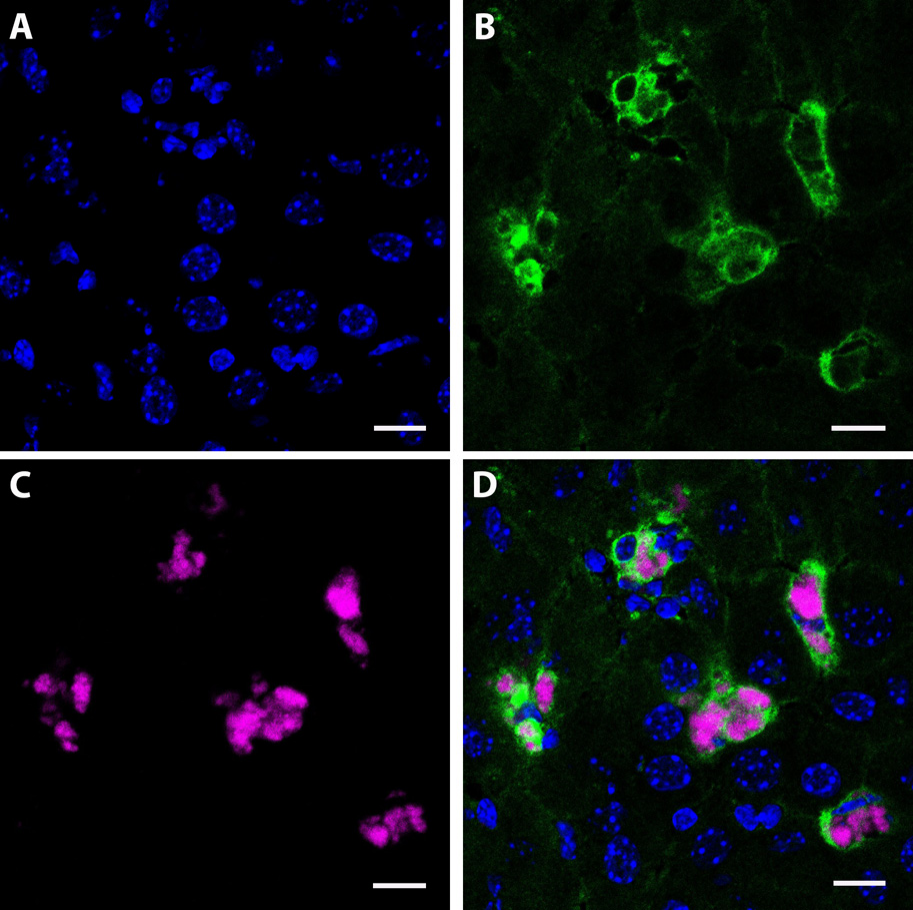
Identification of nanoATV within Kupffer cells. Confocal microscopy images of cryo-sectioned liver collected 12 h after intraperitoneal injection of nanoATV. Immunofluorescence images are as follows: **(A)** Cell nuclei stained with DAPI (blue); **(B)** Macrophages (Kupffer cells) stained with F4/80 and Alexa Fluor 488 (green); **(C)** NanoATV labeled with CF633 (pink); **(D)** Overlay of F4/80-labeled macrophages and CF633-labeled nanoATV. Images were acquired with a LSM 710 confocal microscope at 40x. (scale bars = 10 μm).

**Fig 5:**
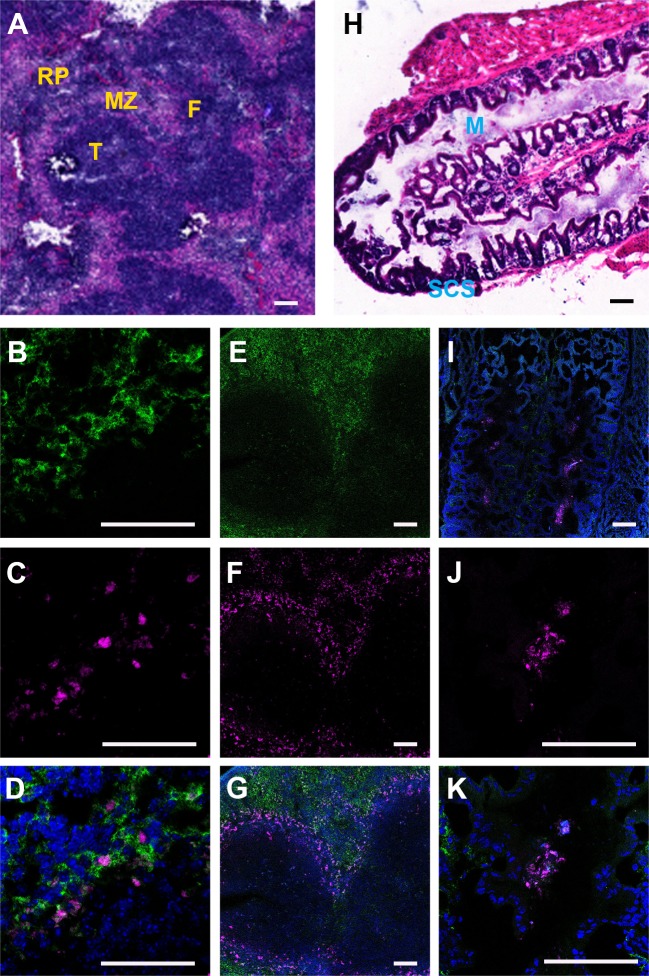
Identification of nanoATV within spleen and lymph node. Confocal microscopy images of cryosectioned spleen and mesenteric lymph nodes collected 12 h after intraperitoneal injection of CF633-labeled nanoATV (pink). Cell nuclei were stained with DAPI (blue); macrophages were stained with Alexa Fluor 488-linked F4/80 antibody (green). Immunostainings as follows: **(A)** H&E of spleen; **(B)** F4/80 labeled splenic macrophages; **(C)** CF633-nanoATV within spleen; **(D)** Overlay of (B) and (C); **(E)** F4/80 labeled splenic macrophages; **(F)** CF633-nanoATV in spleen; **(G)** overlay of (E) and (F); **(H)** H&E of lymph nodes; **(I)** overlay of F4/80 labeled macrophages and CF633-nanoATV in lymph nodes; **(J)** CF633-nanoATV within lymph nodes; **(K)** overlay of F4/80 labeled macrophages and CF633-nanoATV in lymph nodes. Abbreviations: RP (red pulp), MZ (marginal zone), F (B-cell rich), T (T-cell rich), M (medulla), and SCS (subcapsular sinus). Magnifications: 40X for **B, C, D, J** and **K**; 10X for **A, E, F, G, H and I.** (scale bars = 100 μm).

**Fig 6:**
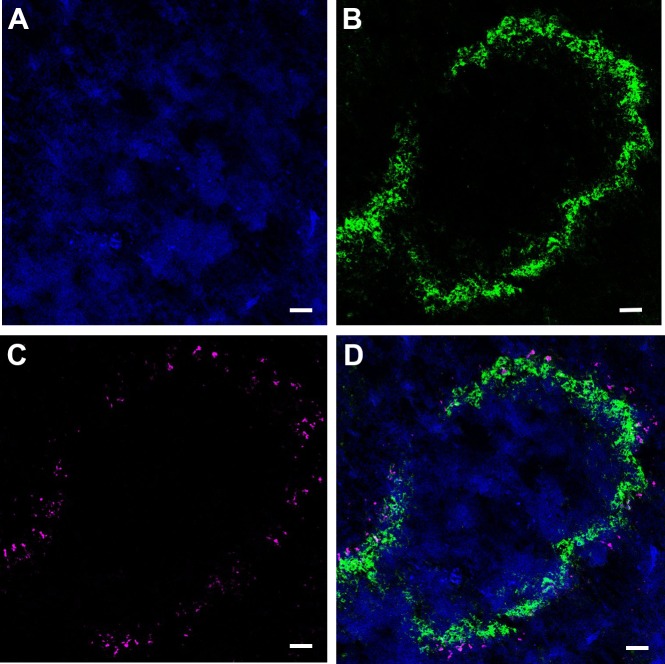
NanoATV within marginal zone macrophages of the spleen. Confocal microscopy images of cryosectioned spleen collected 12 h after intraperitoneal injection of nanoATV. Immunofluorescence images are as follows: **(A)** Cell nuclei stained with DAPI (blue); **(B)** Macrophages stained with CD169 and Alexa Fluor 488 (green); **(C)** NanoATV labeled with CF633 (pink); **(D)** Overlay of CD169-labeled macrophages and CF633-labeled nanoATV. Images were acquired with a LSM 710 confocal microscopy at 10x. (scale bars = 100 μm).

To determine the cellular distribution of nanoATV within tissues, CF633-labeled nanoATV was administered to mice and specific cell markers were used to determine cell depots in liver, spleen, and lymph nodes using confocal microscopy. We chose to assess this at 12 h post injection as that was the time when maximal drug concentrations were detected in these tissues (Fig 3). Cryosections of liver were immunostained for the Kupffer cell marker F4/80 (Fig 4). Individual panels show nuclei (blue, Fig 4A), F4/80+ Kupffer cells (green, Fig 4B), nanoATV alone (pink, Fig 4C), and the merged image (Fig 4D) and demonstrate the presence of nanoATV (pink) in Kupffer cells (F4/80 positive, green). Drug particles were also observed in the medullary sinus of mesenteric lymph nodes and within the spleen along follicular borders (Fig 5). Fig 5B and 5E show F4/80+ stained splenic macrophages (green) and Fig 5C and 5F show accumulation of nanoATV (pink) in spleen. The merged images (Fig 5D and 5G) demonstrate the presence of nanoATV along the borders of splenic follicles. Fig 5I and 5K show localization of nanoATV and F4/80+ cells in mesenteric lymph nodes. Localization of nanoATV was clearly visible in red pulp macrophages, marginal zone macrophages (Fig 5) and marginal metalophillic cells (Fig 6) along the marginal zone of the spleen.

**Fig 7:**
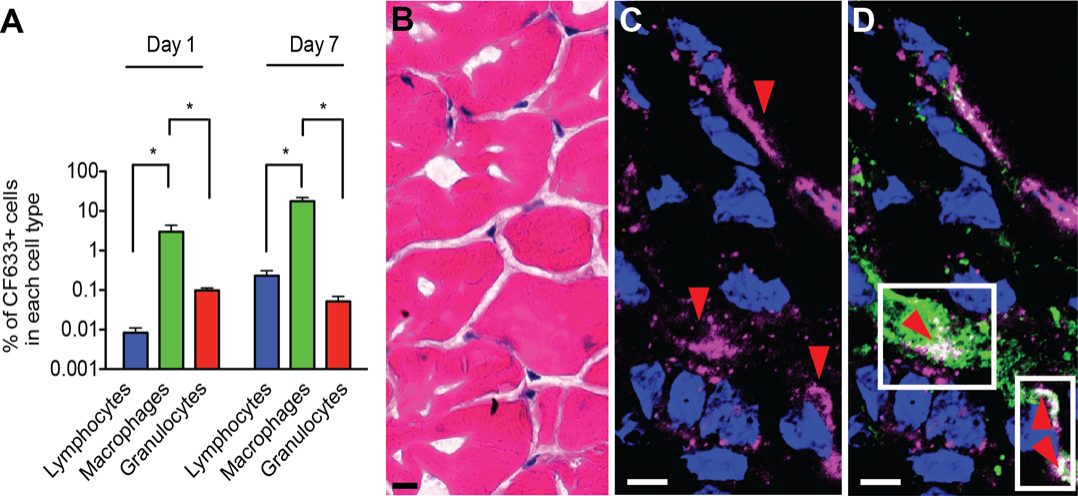
Identification of nanoATV in cells after intramuscular administration. Balb/c mice injected intramuscularly with 100 mg/kg CF633-nanoATV were sacrificed on days 1 and 7 after injection. Cryosections were prepared for H&E staining and for confocal microscopy. **(A)** Flow cytometric analysis of spleen demonstrates significant uptake of nanoATV in macrophages (CD45+CD3-CD11b+F4/80+) compared to lymphocytes (CD45+CD3+) and neutrophils/granulocytes (CD45+CD3-CD11b+F4/80-) at days 1 and 7. Data are represented as mean per mouse ± SEM and considered significant at P<0.05 (Student’s t-test). **(B)** Cryosections of muscle at the site of injection stained with H&E; 40X. **(C)** The muscle cryosection was stained with DAPI for nuclei (blue). The red arrowheads show the accumulation of nanoATV at the site of injection, 63X. **(D)** Macrophages were immunostained with CD68 antibody and Alexa Fluor 488 secondary antibody. Colocalization of nanoATV (pink) and CD68+ cells (green) at the site of injection is indicated with red arrowheads. Nuclei were stained with DAPI (blue), 63X. (scale bars = 10 μm)

Cell and tissue distribution of nanoART was also determined following IM administration, a more clinically relevant dose route. Mice were injected with 100 mg/kg of CF633-labeled nanoATV and tissues collected on days 1 and 7 after injection. CF633+ cells among lymphocytes, macrophages and granulocyte populations in spleen were determined using flow cytometric analysis (Fig 7A and S1 Fig). Tissue macrophages had the highest percent of CF633+ cells with 3.0 ± 1.3% at day 1 and 17.7 ± 4.2% at day 7 compared to lymphocytes and granulocytes (P < 0.05, Student’s t-test). In fact, the percent of CF633+ cells in lymphocytes and granulocytes were negligible and at the limit of quantitation. The gating strategy was based upon CD45, CD3, CD11b and F4/80 cell markers and is demonstrated in S1 Fig. In addition, muscle tissue from the site of injection was cryosectioned and immunostained with anti-CD68 antibody (a macrophage marker). Hematoxylin and eosin staining (Fig 7B) was performed to demonstrate normal histology of muscle following IM injections. Confocal microscopy revealed nanoATV (pink) at the site of injection (Fig 7C) with accumulation and co-localization within CD68+ macrophages (green, Fig 7D).

## Discussion

Together with CD4+ T lymphocytes, macrophages are the main targets for HIV [8, 11, 36]. Macrophages do not undergo virus-induced cytotoxicity and can migrate to virus-infected tissues to participate in HIV dissemination [11, 36]. In the gastrointestinal tract, infected macrophages damage mucosal barriers, resulting in a range of co-infections, a hallmark of HIV type 1 infection [37]. Therefore, ARVs that target macrophages may have therapeutic advantages over those that do not.

**Fig 8:**
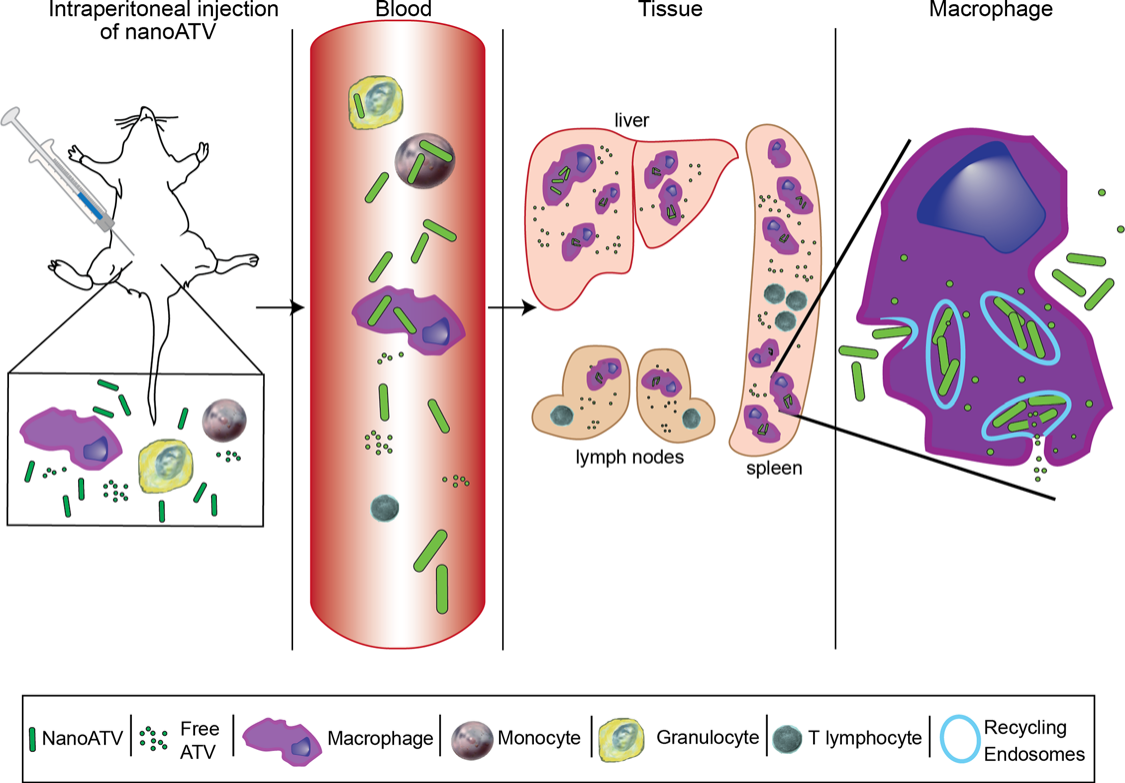
Schematic of nanoATV cellular uptake and tissue distribution. Balb/c mice are injected with nanoATV directly into the peritoneum. This leads to an inflammatory reaction in which primarily granulocytes are induced. The nanoparticles are taken up, principally by macrophages and to a lesser degree by granulocytes. These cells and unincorporated nanoparticles leave the blood to reach Kupffer cells in the liver, splenic macrophages and other phagocytes including those in lymph nodes. In tissue the macrophages encase and store the drug particles in endosomal compartments.

In this study, we demonstrate that MPs are the major cellular drug depot through their ability to quickly engulf drug nanoparticles. This permits the drug to rapidly establish tissue depots. It is plausible that nanoART is trafficked both by MP to regional lymph nodes and tissues and through infiltration from free particles present in plasma. These data importantly, support prior works performed in our laboratories in cultured monocyte-derived macrophages [10, 23, 25, 30]. Moreover, this work demonstrates a role for nanoART to extend drug half-life [23]. The advance, if realized, would benefit both infected people and those at risk for infection; especially those with poor drug adherence or chronic medical conditions such as malabsorption where drug toxicities reduce adherence to mandated drug regimens [9, 12, 38–42]. At a community level, nanoART formulations could improve patient care in regions of the world with limited access to ARV [43]. For those populations, ARV could be administered at extended time intervals with a goal of every 2–6 months [12, 13]. To such ends, the goals of the current study were to uncover the cellular and tissue drug depots of nanoART and their carrying capacities *in vivo*; a summary of the experimental design is provided in Fig 8. These results would provide a first step towards realizing the potential for long acting ART therapies in the care of HIV-infected people.

Prior studies performed in our laboratories and relevant to the current study demonstrated that MP are a major potential site of nanoparticle drug retention [10, 13, 14, 19, 20, 23, 25, 30]. In particular, Kupffer cells were shown to be a nanoART depot compared to other cells in the liver including hepatocytes [13]. However, those studies required isolation of the Kupffer cells and hepatocytes by flow cytometry and subsequent drug quantitation by UPLC-MS/MS. The current study supports such prior results. Direct confocal microscopy identification of the nanoATV cell depot supports the ability of tissue macrophages to serve as both depots and carriers of drug. The presence of drug loaded polymers within the reticuloendothelial system (RES) is well-known and accepted [10, 26, 44–51] and is important as the RES is also the site of viral persistence [8, 11, 52, 53].

While these results demonstrate considerable ARV content in sites of known persistent viral infection, we posit that such findings can be further improved. Indeed, uptake, storage and release of drug by MP can be facilitated even further by optimization of nanoparticle targeting and further amendments to manufacturing conditions such as particle size, shape, charge and polymer choice [24–26]. Moreover, migratory macrophages carrying drug and distributing to other organs would increase the antiretroviral efficacy. As seen in this study, macrophages are the major reservoirs for nanoATV and can traffic to lymph nodes, spleen and liver. The overarching goal is to further facilitate cell-based drug depots in order to decrease drug degradation while concurrently allowing slow ARV release at sites of active viral infection over periods of months. The outcome of such works would result in monthly drug-dosing intervals while still maintaining consistent antiretroviral efficacy and reducing development of viral resistance patterns. These works provide an important step forward towards establishing a framework of manufacturing nanoART with improved PK and pharmacodynamic properties over more traditional antiretroviral approaches.

NanoART within macrophage sorting and recycling endosomes bypasses cellular metabolism [10]. Prior proteomic studies showed that nanoART, but not polymer or free drug, affects functional changes in macrophages that include increased phagocytic, migratory, and secretory capacities resulting in a unique activation state with the potential to facilitate drug depots [30]. *In vivo* in HIV-1 infected humanized mice, nanoART enhanced drug biodistribution with reduced loss of CD4+ T cells and reduced viral loads to undetectable levels [19, 20]. What remained to be elucidated were nanoART’s cellular and tissue drug depots, the carrying capacity for cellular drug sites *in vivo*, and the *in vivo* biological responses to nanoART. We posited that macrophages serve as the major *in vivo* carrier of nanoART with a carrying capacity greater than and/or similar to *in vitro* studies and provide drug depots within lymph nodes, liver, and spleen. Furthermore, we now show that nanoART elicits similar activation without substantial inflammatory responses. MP are the major depots and carriers of nanoART leading to high concentrations in liver and spleen as well as regional mesenteric lymph nodes. Prior work demonstrated that nanoART elicits a unique activation state in macrophages, which is supported by the current *in vivo* studies that provide a functional basis for cell-based carriage and depot formation of the nanoparticles [30].

To determine the cellular and tissue nanoART depots as well as the macrophage carrying capacity we injected the nanoparticles IP and followed biodistribution over 72 h. This included peritoneal cell collections and parallel collection of mesenteric lymph nodes, liver, and spleen to assess drug carriage and distribution kinetics. Amongst the granulocyte and mononuclear cell populations, nanoATV was found preferentially in macrophages. However, other cell types were also shown to contain drug nanoparticles. Whether such alternative drug depots represent drug tightly associated with the cell rather than within the cell is not known. This broader cell association, nonetheless, was not observed after 4 h. Differentiating cells were not affected and macrophages were the principal nanoATV depot at all times tested. In tissue culture, macrophages show rapid uptake of nanoATV (> 30 μg ATV/10^6^ cells within 8 h) [24]. *In vivo*, macrophages took up approximately 11 μg ATV/10^6^ cells and granulocytes retained 1–2 μg ATV/10^6^ cells. This discrepancy may be due to a difference in controlled concentration as occurs *in vitro*. While monocytes initially phagocytized nanoATV, drug was quickly expelled. Thus monocytes may be better suited for carriage rather than storage. Differentiating cells took up the least amount of nanoATV.

Drug concentrations in plasma peaked 1 h after nanoATV injection. This reflected, in measure, the known peak drug concentrations for native oral ATV of around 2.5 h [54]. The change in drug levels could be the result of drug rapidly dissociating from the particles or polymer release. To address this we investigated regional mesenteric lymph node drug distribution. Surprisingly, lymph nodes retained nearly all deposited drug, supporting the idea that the peritoneal macrophages served to carry the drug from the infection site to the regional nodes. How nanoART enters lymph nodes is unclear. Both dendritic cells and macrophages can enter the lymph node typically as part of lymph through afferent lymphatic vessels [55–57] Thus cells carrying drug may enter through lymph and release drug to macrophages found in the medulla or those in the subcapsular sinus. Alternatively, drug may be stored in dendritic cells in the follicle, subcapsular sinus and T zone. Our data suggest that lymph nodes can serve as drug depots with potential to affect delivery to infectious sites and as such to affect drug-dosing intervals. Spleen showed the highest drug levels at ~1000 μg ATV/g tissue at 48 h. While the liver also displayed a high concentration of drug, levels were not retained to the same extent as in spleen and lymph nodes.

The use of zymosan A provided an important control for these studies. The question asked using this known immune modulator was whether immune modulation was required to incite a macrophage activation response necessary for particle uptake and distribution. The answer, notably, was no. Studies in other laboratories performed in mice using zymosan A as an inflammatory agent showed maximal infiltration of granulocytes at 12 h, after which resolution of granulocyte infiltration began [31, 32]. These studies also showed macrophage numbers fell initially at 2 h and continually rose thereafter over 72 h. In our studies, zymosan A induced maximal granulocyte infiltration at 4 h while macrophage numbers initially fell at 4 h. The latter cell population peaked at 12 h and then declined. NanoATV, while not eliciting robust activation, did produce a unique inflammatory response. Granulocyte numbers in mice treated with nanoATV paralleled what was observed with zymosan A. However, macrophage numbers diminished but never recovered, as observed with zymosan A treatment, and peak numbers did not occur until 24 h. Macrophage depletion also occurred following P188 treatment, suggesting that this response is due in part to the polymer, however recovery to initial levels was observed by 72 h. Thus macrophage depletion and failure to recover following nanoATV treatment might be a result of trafficking of nanoATV-loaded macrophages to the regional lymph nodes.

In a replicate experiment, CF633-labeled nanoATV was administered IM to mice at 100 mg/kg (2.5 mg ATV/25 g mouse) to investigate cell and tissue nanoATV distribution. Immunofluorescence staining using the CD68 marker demonstrated significant accumulation of nanoATV in macrophages at the site of injection. This observation serves to support the hypothesis that macrophages serve as drug carriers from the injection site. However, limited accumulation of nanoATV was seen in spleen and liver. This reflected more limited drug accumulation in the RES following IM compared to IP injections. Flow cytometry analysis demonstrated that macrophages preferentially take up drug nanoparticles compared to other cell types (Fig 7A). It should also be noted that the percent of CF633+ macrophages increased on day 7. A limited input to the spleen following a 2.5 mg nanoATV dose/mouse supports the need to use drugs with longer half-lives. Moreover, receptor targeting on macrophages will aide in the process as such particles have already demonstrated substantial improved biodistribution to sites of viral infection [58, 59].

Overall, we demonstrate that the ATV nanoparticles are rapidly engulfed by macrophages and drug concentrations appear rapidly in the regional lymph nodes and RES. A recent study demonstrated the significance of high plasma drug levels in preventing HIV-1 infection in NOD/SCID/γc^−/−^/bone marrow/liver/thymus (BLT) humanized mouse model when administered as a pre-exposure prophylaxis regimen [60]. A single dose of 15 mg rilpivirine long-acting nanoformulation per mouse prevented early infection. However, once the plasma drug levels decreased, the mice were no longer resistant to infection. This shows the necessity for tissue drug depots, especially in sites of viral infection that could stop viral replication. The PLGA rilpivirine long-acting nanoformulation achieved the desired antiretroviral effects, however high doses were needed likely due to limited drug loading capacity of PLGA nanoparticles (10–15%). This was seen despite a drug half-life of ~50 hrs. The poor drug distributions were likely secondary to lack of tissue depots beyond the site of injection. Presence of tissue drug depots is vital for sustained plasma levels and improved antiretroviral efficacy at later time points. In fact, our laboratory showed significant biodistribution of nanoART in spleen, lymph node and other viral infection sites along with improved antiretroviral efficacy for up to 4 weeks with doses of less than 1.125 mg per mouse using targeted nanoformulations of cabotegravir (our unpublished observations). Importantly, in the present study, we demonstrated that the major source of drug in tissue depots is cell-based P188-nanoATV. The rapid carriage of nanoATV to sites of viral infection clearly demonstrates the potential for delivery of ART to viral reservoirs, thus delivering ART to CD4+T-cells in infected individuals.

In conclusion, our results lend credence to macrophages as the major cellular drug depot with granulocytes serving as minor sources for drug carriage. Moreover, both MP and granulocytes may traffic drug to the periphery where tissue depots are first established in mesenteric lymph nodes. Lymph nodes, spleen, and liver are tissue sites for long-term drug depots. NanoATV does elicit an activation state to potentiate drug particle carriage and dissemination. Taken together, these observations support the role of macrophages as conductors for drug trafficking and as protectors of drug from elimination.

## Supporting Information

S1 Fig Flow cytometric analysis of spleen cells for identification of CF633+ cells. Balb/cJ mice were administered 100 mg/kg of CF633-labeled nanoATV intramuscularly and sacrificed at days 1 and 7. Single cell suspensions of spleen were stained with fluorochrome-labeled antibodies; PE-Cy7-CD45, FITC-CD3, PE-CD11b and BV421-F4/80. CD45^+^CD3^+^ cells were considered as lymphocytes, the CD45^+^CD3^–^CD11b^+^F4/80^+^ population was considered as macrophages and CD45^+^CD3^–^CD11b^+^F4/80^–^ cells were gated as the neutrophil/granulocyte population. Image demonstrating gating for mice treated with **(A)** nanoATV or **(B)** untreated and sacrificed at day 7 following treatment.
(TIF)
