# Supplementary figures and images for: Cellular Responses and Tissue Depots for Nanoformulated Antiretroviral Therapy
Source: PLoS One. 2015 Dec 30;10(12):e0145966. doi: 10.1371/journal.pone.0145966 (PMC4696780; doi:10.1371/journal.pone.0145966)

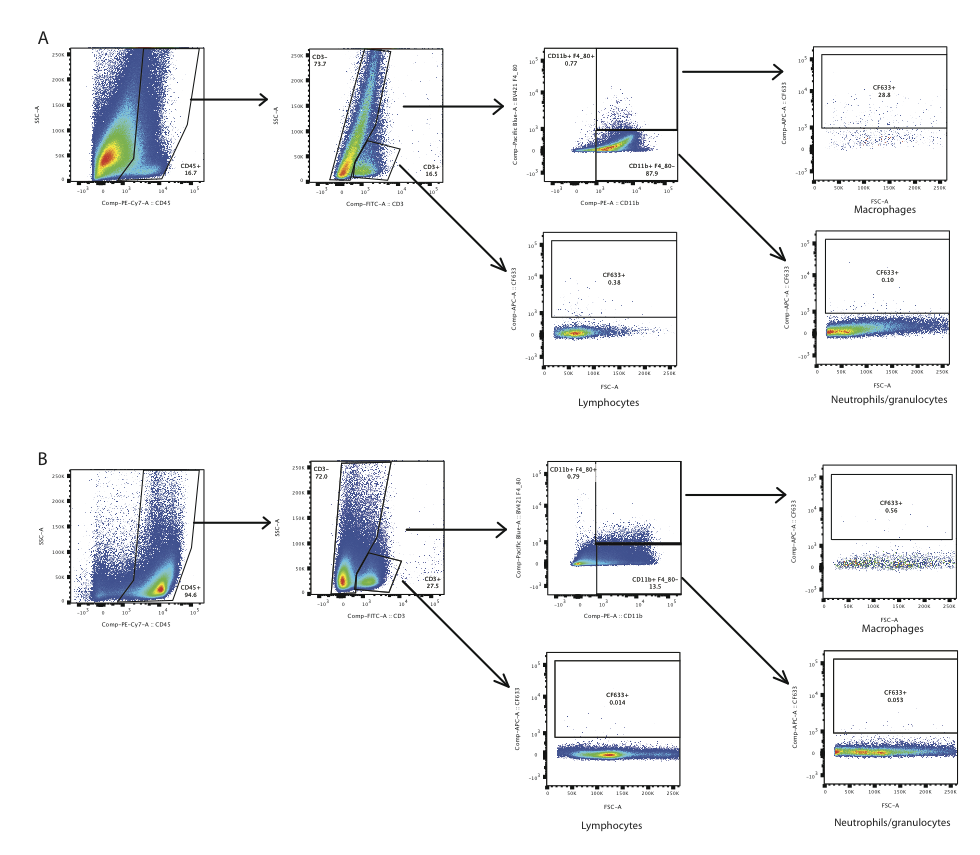

Supplement: S1 Fig — Balb/cJ mice were administered 100 mg/kg of CF633-labeled nanoATV intramuscularly and sacrificed at days 1 and 7. Single cell suspensions of spleen were stained with fluorochrome-labeled antibodies; PE-Cy7-CD45, FITC-CD3, PE-CD11b and BV421-F4/80. CD45+CD3+ cells were considered as lymphocytes, the CD45+CD3–CD11b+F4/80+ population was considered as macrophages and CD45+CD3–CD11b+F4/80– cells were gated as the neutrophil/granulocyte population. Image demonstrating gating for mice treated with (A) nanoATV or (B) untreated and sacrificed at day 7 following treatment. (TIF) [file pone.0145966.s001.tif]
